# *Clostridium acetobutylicum* Biofilm: Advances in Understanding the Basis

**DOI:** 10.3389/fbioe.2021.658568

**Published:** 2021-06-03

**Authors:** Huifang Zhang, Pengpeng Yang, Zhenyu Wang, Mengting Li, Jie Zhang, Dong Liu, Yong Chen, Hanjie Ying

**Affiliations:** ^1^State Key Laboratory of Materials-Oriented Chemical Engineering, College of Biotechnology and Pharmaceutical Engineering, Nanjing Tech University, Nanjing, China; ^2^School of Chemical Engineering and Energy, Zhengzhou University, Zhengzhou, China

**Keywords:** *Clostridium acetobutylicum* biofilm, extracellular matrix, physiological changes, non-classically secreted proteins, heteropolysaccharides

## Abstract

*Clostridium acetobutylicum* is an important industrial platform capable of producing a variety of biofuels and bulk chemicals. Biofilm of *C. acetobutylicum* renders many production advantages and has been long and extensively applied in fermentation. However, molecular and genetic mechanisms underlying the biofilm have been much less studied and remain largely unknown. Here, we review studies to date focusing on *C. acetobutylicum* biofilms, especially on its physiological and molecular aspects, summarizing the production advantages, cell physiological changes, extracellular matrix components and regulatory genes of the biofilm. This represents the first review dedicated to the biofilm of *C. acetobutylicum*. Hopefully, it will deepen our understanding toward *C. acetobutylicum* biofilm and inspire more research to learn and develop more efficient biofilm processes in this industrially important bacterium.

## Introduction

Biofilms are the main living structure of microorganisms in nature. They are closely related to our lives. Traces of biofilms can be found in living tissues, medical devices, and industrial settings. It is estimated that more than 90% of bacteria can form this special structure, which makes biofilms a rich and developable biological resource.

**FIGURE 1:**
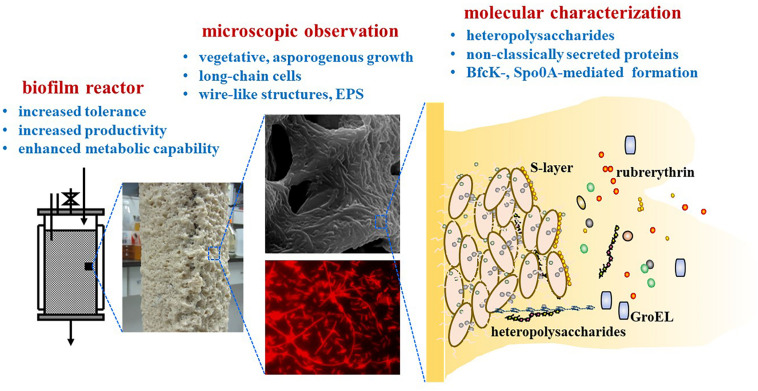
Characterized aspects of *C. acetobutylicum* biofilm.

In recent years, more and more biofilms are engineered as cell factories for biological manufacturing. A canonical example is the biofilm of the solvent-producing *Clostridium*, which is an important industrial platform and widely employed for biological production of acetone-butanol-ethanol (ABE) and a variety of other important chemicals. As early as 20 years ago, adsorption and immobilization of *C. beijerinckii* on clay bricks as biofilm was reported which greatly improved the ABE fermentation efficiency (Qureshi et al., 2000). In recent years, developments of *Clostridium* biofilms for improved production efficiency are extensively reported. However, compared with the biofilms of other industrial microbes like *Escherichia coli*, *Saccharomyces cerevisiae* and *Bacillus subtilis*, little has been known about the molecular mechanisms underlying the *Clostridium* biofilms. The physiological responses, growth changes, the biofilm composition and its genetic determinants, remain to be further understood. In view of this, we review studies focusing on *C. acetobutylicum* biofilms, especially its physiological and molecular aspects (Figure 1). Although there is a review regarding pathogenic clostridia (Pantaleon et al., 2014), this will represent the first review in the field of industrial *C. acetobutylicum* biofilms. While pathogenic clostridia such as *C. difficile* and *C. perfringens* typically form multi-species biofilm in human gut, they can also form mono-species biofilm on abiotic surfaces like industrial *C. acetobutylicum*. Pathogenic clostridia do share many common characteristics with *C. acetobutylicum*. For example, they all sporulate, possess flagellar motility, and have a master transcription regulator Spo0A, which are important aspects involved in biofilm formation (Dawson et al., 2012; Ðapa et al., 2013; Dapa and Unnikrishnan, 2013). However, compared to *C. acetobutylicum*, pathogenic clostridia do not accumulate excessive products and are less studied from a metabolic perspective. Biofilms of pathogenic clostridia were often studied in medical settings in terms of biofilm growth and cell survival over a relative short time period. By contrast, *C. acetobutylicum* biofilm were often studied in bioreactors over hundreds of hours during continuous fermentation. So, research aims and methodology for industrial *Clostridium* biofilm can be quite different from those for pathogenic clostridia. Factors considered important in pathogenic clostridia may not hold for *C. acetobutylicum* and vice versa. Comparison of biofilm between *C. acetobutylicum* and pathogenic clostridia or other industrial clostridia is included in this review when necessary. Hopefully, this review will deepen our understanding of *C. acetobutylicum* biofilm, and inspire more research to learn and develop more efficient *C. acetobutylicum* biofilm processes that can become industrial models of biofilm application.

## Physiological Changes of Cells in the Biofilm

### Vegetative and Asporogenous Growth

Spore formation is a common physiological characteristic of *C. acetobutylicum*. It is generally believed that *C. acetobutylicum* initiates the expression of spore-producing genes when entering stationary phase. Early studies found that spore formation and solvent production occurred simultaneously. So, sporulation and solventogenesis were widely considered to be coupled (Lütke-Eversloh and Bahl, 2011). However, this seems not true in the biofilm. Liu et al. (2018) found that the spores of *C. acetobutylicum* in biofilm were reduced over time during long-term continuous fermentation. Finally, *C. acetobutylicum* biofilm cells seemed to eliminate sporulation and performed vegetative growth. This suggested that the cells in biofilm could maintain an active metabolic state instead of being in dormancy, leading to extended cell lifespan and prolonged productivity. Temporal transcriptomics analysis confirmed that expression of the genes responsible for spore formation was apparently down-regulated in the biofilm over time. The gene encoding the sporulation regulator σK (sigK, CA_C1689) was down-regulated over time up to eightfold. The genes involved in spore coat synthesis (CA_C0613-0614, CA_C1335, CA_C1337-1338, CA_C2808-2910, and CA_C3317) and the operon CA_C2086-2093 involved in stage III sporulation were decreased over time by 6–24-fold. The most down-regulated genes were those encoding the small, acid-soluble proteins (SASP) that are used to coat DNA in spores (CA_C1487, CA_C1522, and CA_C2365), which were significantly down-regulated by 48–200-fold. It is generally believed that the solvent production in *C. acetobutylicum* is coupled to the formation of spores, but the biofilm shows that *C. acetobutylicum* can greatly increase the solvent production without sporulation. This decoupling is also evidenced by other studies (Jones et al., 2011). Sporulation is also of particular interest in pathogenic clostridia because it is thought to cause persistent infections. Interestingly, elimination of sporulation also appeared in a study of *C. difficile* biofilm (Ðapa et al., 2013). In this study, the spore number in *C. difficile* biofilm on day 3 and 5 was extremely low (0.0001%) compared to the control (40–50%). However, other studies showed that the spore number within a 6 days old biofilm varied from 10% to more than 60% depending on the *C. difficile* strains (Dawson et al., 2012; Semenyuk et al., 2014). Different with these pathogenic strains, the biofilm of *C. acetobutylicum* was developed in a long-term fermentation process with continual nutrient supply. This might allow continual cell growth and reduce stress-induced sporulation.

### Long-Chain Cells

Besides elimination of sporulation, *C. acetobutylicum* biofilm cells also exhibited significant morphological changes. In traditional planktonic cell culture, cells are dispersed and short rod-shaped. However, cells became longer in the biofilm and were often linked in chain-like structure. Interestingly, the elongated chain-like morphology was also observed for *B. subtilis* biofilm cells. In *B. subtilis*, a transcriptional regulator SinR represses the genes responsible for EPS production and promotes cell motility and separation. During the development of biofilm, SinR activity is decreased and leads to EPS production and loss of cell motility. As a result, motile single cells turn to long chains of non-motile cells (Lemon et al., 2008). *C. acetobutylicum* also has a SinR homolog, but whether the SinR can also cause the long chain of cells in *C. acetobutylicum* biofilm remains to be verified. Similarly, elongated rod morphology was observed for *C. tyrobutyricum* after long-term adaptation in a biofilm reactor (Jiang et al., 2011), and also observed for *C. thermocellum* showing that vegetative cells attached on cellulosic fibers formed progressively longer chains over time (Dumitrache et al., 2013).

### Modulated Gene/Protein Expression

Liu et al. (2016) carried out many transcriptomics and proteomics studies of *C. acetobutylicum* biofilm cells and revealed more unique phenotypes of the biofilm cells. They compared transcriptomic differences between free cells and biofilm cells during batch fermentation, studied transcriptomic changes in biofilm cells during long-term repeated batch fermentation (Liu et al., 2018), and performed proteomic analysis of biofilm (Liu et al., 2018; Yang et al., 2020). Genes/proteins that are often differentially regulated in *C. acetobutylicum* biofilm are summarized in Table 1. Results showed that 16.2% of genes in *C. acetobutylicum* genome were differentially expressed in the biofilm. The most dramatic changes occurred to amino acid biosynthesis, with sulfur uptake and cysteine biosynthesis genes being the most up-regulated and histidine biosynthesis genes being the most down-regulated. *C. acetobutylicum* biofilm cells also up-regulated iron and sulfur uptake genes, Fe-S cluster biosynthesis genes as well as glycolysis genes, which could probably account for their increased metabolic activity. In addition, granulose formation and extracellular polymers metabolism were also significantly modulated. Cell motility, chemotaxis and flagella biosynthesis were gradually down-regulated in the biofilm during the long-term fermentation process. By contrast, central metabolism such as glycolysis, solvent synthesis and sugar utilization was relatively stable during the long-term biofilm fermentation. Similarly, *C. thermocellum* biofilm cells showed greater expression of genes involved in central metabolism such as glycolysis, H_2_ production and ATP regeneration, while relatively lower expression of genes involved in flagellar motility and chemotaxis (Dumitrache et al., 2017). This could explain why cells can maintain long-term metabolic activity in the biofilm. Overall, these studies provide valuable insights into the molecular basis of *C. acetobutylicum* biofilm and should be very useful for understanding and developing biofilm-based processes.

**TABLE 1:** Some genes/proteins differentially regulated in *C. acetobutylicum* biofilms*.

Genes/proteins	General change	Description
**Glycolysis**		
CA_C0570 (glcG)	Up-regulated	PTS sugar transporter subunit
CA_C3657 (gapN)	Up-regulated	NADP^+^-dependent Glyceraldehyde-3-phosphate dehydrogenase
CA_C0712 (pgm)	Up-regulated	Phosphoglycerate mutase
CA_C0713 (eno)	Up-regulated	Enolase
CA_P0141-0142, CA_C0809-0810	Up-regulated	Hydrogenases and maturation factors
CA_C3552 (ldh)	Up-regulated	L-lactate dehydrogenase
CA_C2967 (alsD)	Down-regulated	α-Acetolactate decarboxylase
**Pentose metabolism**		
CA_C1339-1341 (araEAD)	Up-regulated	L-arabinose utilization
CA_C2610, CA_C3451	Up-regulated	D-xylose utilization
CA_C1343 (xfp)	Up-regulated	Phosphoketolase
CA_C1347-1348 (tkt, tal)	Up-regulated	Pentose phosphate pathway
**Polysaccharides metabolism**		
CA_C2383, CA_C0358	Up-regulated	Xylanase/chitin deacetylase
CA_C0804, CA_C1968	Up-regulated	Pectate lyase
CA_C0910	Up-regulated	Cellulosomal scaffolding protein
**Iron and sulfur assimilation**		
CA_C0788-0791, CA_C1029-1030	Up-regulated	Iron assimilation
CA C0102-0110	Up-regulated	SULFATE assimilation
**Sporulation and chemotaxis**		
CA_C2086-2093, CA_C2859 (spoIIID)	Up-regulated at early stages then down-regulated over time	Stage III sporulation proteins
CA_C1487, CA_C2365	Down-regulated	Small acid-soluble spore protein
CA_C2908-2910, CA_C1337-1338	Down-regulated	Spore coat protein
CA_C0117	Up-regulated	CheY-like chemotaxis protein
CA_C2745, CA_C2419, CA_C2803	Down-regulated	Methyl-accepting chemotaxis protein
CA_C2205 (fliD)	Down-regulated	Flagellar hook-associated protein
**Oxidative stress response**		
CA_C1547-1549	Up-regulated at early stages then down-regulated over time	Thioredoxin and thioredoxin reductase
CA_C1570-1571	Up-regulated at early stages then down-regulated over time	Glutathione peroxidase
CA_C3597-3598	Up-regulated at early stages then down-regulated over time	Reverse rubrerythrin
**Others**		
CA_C0252-0255	Up-regulated	Nitrogen-fixation
CA_C0280-0281	Up-regulated	Molybdate transporter
CA_C3634-3644	Up-regulated	Oligopeptide transporter
CA_C1447, CA_C1504, CA_P0109, CA_P0128	Down-regulated	MDR-type permease

*Results are summarized from the studies of Liu et al. (2016, 2018) and Yang et al. (2020).

## Extracellular Matrix of the Biofilm

### EPS and Wire-Like Structures

Biofilm is composed of water, EPS, cells, etc. The EPS is mainly secreted by cells or derived from cell lysis, mainly containing biological macromolecules such as polysaccharides, proteins, nucleic acids, lipids, etc. These components can play important roles in the structure of the biofilm.

Unlike *C. thermocellum* that forms a distinctive monolayer biofilm with no extracellular polymeric matrix (Dumitrache et al., 2013), extracellular polymers are clearly found in *C. acetobutylicum* and many other clostridia. The EPS was so abundant that it often could be visibly observed. Liu et al. (2013) observed a thick and sticky layer of biofilm matrix stacked on the carrier surface. Through SEM observation they found that the cells in the biofilm of *C. acetobutylicum* were present in aggregates and covered by a large number of EPS they produced, forming a multiplayer biofilm. Later Zhuang et al. (2016) observed the extracellular sheet-like EPS with a transmission electron microscope (TEM), and found it was accumulated around the cells during the formation of the biofilm. Recently, Liu et al. (2018) further observed wire-like structures in *C. acetobutylicum* biofilm under fluorescence microscope. The wires were as long as 50 μm and could be cross-connected and imbedded in cell aggregates. Yang et al. (2020) also found wire-like structures that cross-linked cells in *C. acetobutylicum* biofilm. These structures disappeared when *spo0A* was disrupted (Yang et al., 2020). Similarly, Engel et al. (2019) found in a bioelectrochemical system that *C. acetobutylicum* cells in the biofilm formed high number and density of filamentous appendages more than 20 μm in length.

### Extracellular Polysaccharides

Extracellular polysaccharide is one of the most common components in biofilms. In many microbes, extracellular polysaccharides play an important role in the formation of biofilms. Mutant strains with defective extracellular polysaccharide synthesis have greatly reduced ability to form biofilms or even produce no biofilms (Danese et al., 2000). Polysaccharide biosynthesis genes are usually located in operons or gene clusters, encoding proteins responsible for repeat unit synthesis, polysaccharide chain initiation and elongation, chain length control, transmembrane transport and process regulation. In addition to the cell wall peptidoglycan biosynthesis genes, there are at least 4 long gene clusters consisting of 10–15 genes (CA_C3059-CA_C3045, CA_C3073-CA_C3060, CA_C2337-CA_C2325, and CA_C2321-2312) in *C. acetobutylicum* genome that potentially make extracellular polysaccharides. Studies have shown that *C. acetobutylicum* can produce extracellular polysaccharides. An early study showed that an extracellular polysaccharide was produced by *C. acetobutylicum* resting cells during a biocatalysis process, which made the reaction system very viscous. According to the metabolic analysis, it was speculated that the polysaccharide might be acetylate glucose polymers (Häggström and Förberg, 1986). Interestingly, a recent study also found a large quantity of acetylated glucose polymers in *Clostridioides difficile* 630Δerm, which were produced by the operon CDIF630erm_02794—CDIF630erm_02798. Bioinformatics predicted that *C. acetobutylicum* had a similar operon CA_C1561-CA_C1565 (Dannheim et al., 2017). However, whether this operon is involved in the secretion of acetylated glucose polymers in *C. acetobutylicum* remains to be verified.

Wallenius et al. (2016) extracted exopolysaccharides from *C. acetobutylicum* chemostat culture under glucose limited condition and characterized them on monosaccharide level through nuclear magnetic resonance (NMR) spectroscopy. The polysaccharides were mainly composed of 40% (molar ratio, the same hereinafter) rhamnose, 34% glucose, 13% mannose, 10% galactose, and 2% arabinose. Liu et al. (2018) extracted polysaccharides in *C. acetobutylicum* biofilm which were then separated by gel chromatography, degraded, derivatized, and analyzed by liquid chromatography. They identified three different polysaccharides SM1, SM2, and SM3. SM1 represented the largest fraction (53%, w/w) in the polysaccharide extract, followed by SM2 (26%, w/w) and SM3 (21%, w/w). Analysis showed that all the three polysaccharides were heteropolysaccharides with glucose as the primary component. SM2 and SM3 shared similar monosaccharide type and molar ratio, consisting of glucose (47–53%, molar ratio, the same hereinafter), mannose (13–15%), rhamnose (10–16%), galactose (9–10%), glucosamine (7–9%) and ribose (4–5%). Compared with SM2 and SM3, SM1 polysaccharide consisted of more glucose (58%), mannose (21%), and glucosamine (13%), but much less rhamnose (0.8%), galactose (0.8%), and ribose (0.4%). SM1 also consisted of unique galacturonic acid (5.5%). The presence of galacturonic acid explained why the *C. acetobutylicum* biofilm substances was alkali-soluble during the extraction process (Liu et al., 2018). Recently, Dong et al. (2020) also used fluorescent lectins to probe for specific sugars during *C. acetobutylicum* biofilm development, and found that fucose and galactose were present in the biofilm.

### Non-classically Secreted Proteins

Extracellular protein has been demonstrated to be another vital component of many bacterial biofilms. In *B. subtilis*, the TasA, and BslA proteins have been reported to be required for the formation of air–liquid or solid–air interface biofilms. In *Staphylococcus aureus*, the Bap protein at the cell surface facilitates cells attachment to the substrate and cell-to-cell interactions (Obana et al., 2020). Research demonstrated that the protein in the biofilm of *C. acetobutylicum* could reach more than 50% (w/w) of the biofilm matrix. However, no biofilm specific proteins such as TasA and BslA have been found in *C. acetobutylicum* so far. Liu et al. (2018) analyzed the proteomics of the biofilm by LC-MS/MS, two-dimensional SDS-PAGE and MALDI-TOF/TOF mass spectrometry, wherein S-layer protein was found one of the most abundant proteins in the biofilm. S-layer is a crystalline monomolecular assembly of proteins. It is a common outermost cell envelope structure in bacteria (Lai et al., 2003; Plaut et al., 2014; Soavelomandroso et al., 2017). In *C. difficile*, S-layer was demonstrated essential for biofilm formation perhaps because it was essential for anchoring proteins required for biofilm formation (Ðapa et al., 2013). In addition to the S-layer protein, a large number of proteins that were expected to be intracellular were also present abundantly in the *C. acetobutylicum* biofilm matrix. GroEL and ruberythrin were two notable examples and they could also be detected in the fermentation supernatant. Proteins released from intracellular pool through unknown secreted pathways have been defined as non-classically secreted proteins. GroEL and rubrerythrin play a canonical role inside cells in correct folding of proteins and adaptation to oxidative stress, respectively. However, there is increasing evidence that non-classically secreted proteins are multifunctional proteins and many of them can moonlight as adhesins (Kainulainen and Korhonen, 2014). In particular, GroEL has often been found involved in cell adherence and biofilm formation in different species (Hennequin et al., 2001; Arai et al., 2015; Arora et al., 2017). Some studies found GroEL was also able to form amyloid-like fibrils under physiological conditions (Chen et al., 2012, 2014), a structure usually important for biofilm formation. Besides GroEL and rubrerythrin, many other non-classically secreted proteins such as the molecular chaperone GroES, elongation factor Tu (Ef-Tu), trigger factor, glyceraldehyde-3-phosphate dehydrogenase (GAPDH), and electron transfer flavoprotein β-subunit were also abundant in the biofilm of *C. acetobutylicum* (Liu et al., 2018). While these non-classically secreted proteins were likely to participate in the formation of biofilm, they were also likely to participate in cellular metabolism because many of them were central metabolic enzymes. Strikingly, these enzymes included almost all the important enzymes for solventogenisis, with the electron transfer flavoprotein (EtfAB), crotonase (Crt), acetoacetyl-CoA: acetate/butyrate CoA-transferase (CtfAB), phosphate butyryltransferase (Ptb), pyruvate:ferredoxin oxidoreductase (Pfor), butyrate kinase (Buk), acetyl coenzyme A acetyltransferase (Thl), acetoacetate decarboxylase (Adc) and alcohol dehydrogenase E (adhE) being the Top 30 abundant extracellular proteins in the biofilm matrix (Liu et al., 2018). However, it is currently unknown how these proteins are secreted outside the cells, and their specific effects on the biofilm of *C. acetobutylicum* need to be studied in detail.

## Production Advantages of Biofilm

### Increased Cell Tolerance

Butanol is toxic to cells. The growth and fermentation of solvent-producing Clostridium such as *C. acetobutylicum* and *C. beijerinckii* are easily inhibited by butanol. In traditional batch fermentation by free cells, the fermentation will cease when butanol titer reaches around 13 g/L, and the total titer of ABE is usually around 20 g/L. At the same time, the fermentation speed is slow, and the fermentation time is usually 60–80 h, with the total solvent productivity only about 0.3 g/L/h. Biofilm increases cellular density inside it, and the highly hydrated extracellular polymeric substances (EPS) of biofilm can act as a protective barrier and timely excrete waste metabolites (Flemming et al., 2016). Therefore, it provides a beneficial microenvironment for cell survival. Thus, the cell viability and environmental adaptability could be improved. Liu et al. (2013) demonstrated that the *C. acetobutylicum* biofilm formed on fibrous matrix significantly increased the butanol resistance of the cells. The survival of the biofilm cells exposed to 15 g/L of butanol for 2 h was 3 orders of magnitude higher than that of the planktonic cells. Later on, they revealed using confocal laser scanning microscope that EPS acted as a barrier to limit the transfer of harmful substances and diluted their concentrations. In addition, cells in different regions of the biofilm displayed different tolerance to harsh environment. Some cells with higher tolerance could continue growth in the biofilm. This heterogeneity of biofilm might be the primary tolerance mechanisms associated with biofilm (Zhuang et al., 2016). The increase in solvent tolerance of *C. acetobutylicum* biofilm cells probably was also associated with their morphological changes. Since the cells in biofilm turned into long-chain cells, compared to the short-rod shape this would decrease their surface-to-volume ratio. As the cell surface and cell membrane are the major targets for the action of organic compounds, many studies have shown that cells of larger size or smaller specific surface area could be more advantageous under toxic solvents (Neumann et al., 2005; Si et al., 2016; Gupta et al., 2020). It should be noted that while the biofilms increase cell tolerance, it could also become an unfavorable situation under certain circumstance. For example, the heterogeneity of biofilm and EPS protection may void antibiotic selection pressure, and thus make fermentation less efficient with genetically engineered strains that require antibiotics to maintain target genes. A solution to this problem is to integrate target genes into *C. acetobutylicum* genome, which can avoid use of antibiotics and will provide long-term expression of target genes.

### Increased Productivity

Performing fermentation using cells in the form of biofilm can greatly improve the productivity (Table 2). Qureshi et al. (2000) demonstrated that *C. beijerinckii* biofilm formed on clay bricks could be used for continuous ABE fermentation. At a dilution rate of 2.0 h^–1^, a total solvent productivity of 15.8 g/L/h was reached, which was approximately 50 times the productivity of free cell batch fermentation. This is the highest productivity ever reported for butanol fermentation. Similarly, Huang et al. (2004) used *C. acetobutylicum* biofilm for continuous fermentation in a fiber bed reactor. The fermentation was continuously performed for more than 600 h, and the best butanol productivity of 4.6 g/L/h was obtained at a dilution rate of 0.9 h^–1^. Continuous fermentation significantly improves productivity, but there is usually a problem of low product concentration. To address this problem, Liu et al. (2013) developed a repeated batch fermentation process based on *C. acetobutylicum* biofilm. The fermentation period was shortened from the traditional 72 to 12–14 h per batch, which is the shortest batch fermentation time yet reported. Unlike the continuous fermentation, glucose could be completely consumed in the repeated batch fermentation. As a result, a high butanol titer of 15.6 g/L was achieved with a solvent productivity of 1.88 g/L/h. This final butanol titer was 21% higher than that of planktonic cells, which could be explained by the improved butanol tolerance of the biofilm cells. On the other hand, the vegetative and asporogenous growth of biofilm cells could maintain an active metabolic state and help to extend cell lifespan. This should contribute a lot to the increased reaction speed and accelerated fermentation process. Furthermore, biofilms can function as a reservoir of enzymes either extracellular or released from cytoplasm (Flemming and Wingender, 2010). Many of the non-classically secreted proteins in *C. acetobutylicum* biofilm matrix were involved in central metabolism. Thus, the extracellular presence of these metabolic enzymes might also help to increase substrate utilization and conversion speed during the fermentation.

**TABLE 2:** Comparison of representative fermentation results obtained from *C. acetobutylicum* biofilm or planktonic cells.

Culture	Process mode	Butanol titer (g/L)	Solvent yield(g/g)	Solvent productivity (g/L/h)	References
*C. acetobutylicum* B3	Free cells	11.8	0.34	0.25	Liu et al., 2014
*C. acetobutylicum* ATCC 55025	Biofilm-immobilized cells, continuous fermentation	∼5	0.42	4.6	Huang et al., 2004
*C. beijerinckii* BA101	Biofilm-immobilized cells, continuous fermentation	∼5	0.38	15.8	Qureshi et al., 2000
*C. beijerinckii* BA101	Biofilm-immobilized cells, batch fermentation	20.9	0.41	0.59	Chen and Blaschek, 1999
*C. beijerinckii* NCIMB 8052	Biofilm-immobilized cells, continuous fermentation	13.4	0.44	0.40	Lee et al., 2008
*C. acetobutylicum* B3	Biofilm-immobilized cells, batch fermentation	15.6	0.38	1.88	Liu et al., 2013

In addition to increasing cell tolerance and productivity, the biofilm also realizes solid-liquid separation and thus is good for product recovery. This makes biofilm fermentation more suitable for process integration. For example, it can be coupled with the product separation process based on resin adsorption or gas stripping. In this way the productivity and equivalent product concentration could be further dramatically improved (Xue et al., 2013; Liu et al., 2014).

### Enhanced Metabolic Capability

Studies have also shown that *C. acetobutylicum* biofilm exhibits enhanced metabolic capabilities, such as the utilization of xylose. The utilization of xylose by cells is of great significance to the utilization of lignocellulose as cheap fermentation feedstock. *C. acetobutylicum* is capable of utilizing xylose, but the xylose utilization is relatively inefficient in the presence of glucose, due to the well-known Carbon Catabolite Repression (CCR) effect. Chen et al. (2013) used pretreated cotton towels to support *C. acetobutylicum* biofilm. They showed that the biofilm cells significantly improved the xylose utilization rate. The xylose utilization rate on pure xylose medium was increased by 25.9%, and on glucose-xylose mixture was increased by 70%, compared to those of free cells, respectively. A subsequent transcriptomic study of *C. acetobutylicum* biofilm found that most of the genes involved in xylose and arabinose catabolism were slightly down-regulated at the initial growth stage, but significantly up-regulated throughout the subsequent stages. This was consistent with the experimental results that the xylose utilization capacity of the surface-adsorbed biofilm cells was improved (Liu et al., 2016). In addition, the transcriptomic study also showed that many genes involved in hydrolysis of xylan, chitin, starch and pectate as well as genes involved in cell wall recycling were apparently up-regulated, especially at the late growth stage (Liu et al., 2016). Since the *C. acetobutylicum* biofilm matrix contains heteropolysaccharides composed of various sugars, the enhanced metabolism of sugars may help the cells to derive energy from these extracellular heteropolysaccharides when nutrients are depleted. Potentially, the enhanced metabolism of extracellular sugars could also help to build and restructure the biofilm matrix.

It should be noted that while metabolic capability can be enhanced in biofilm cells, the metabolic fluxes may be shifted and this could lead to undesirable product pattern. For example, transcriptomic analyses showed that expression of hydrogenases was up-regulated in *C. acetobutylicum* and *C. thermocellum* biofilm cells (Liu et al., 2016). Consistently, the biofilm cells appeared to evolve more H_2_ and produced 37% more acetone as a result of redox balance (Liu et al., 2013). For strains like *C. tyrobutyricum* that produces H_2_ as a target product, this can be a very favorable result (Jo et al., 2008). However, for *C. acetobutylicum* this will cause a decreased butanol selectivity during the ABE fermentation. In this case, metabolic regulation can be considered. Using an exogenous electron carrier to redirect the electron flow toward butanol biosynthesis instead of H_2_ evolution could greatly improve the butanol selectivity of the biofilm cells (Liu et al., 2013).

## Influencing Factors and Regulatory Genes

### Biofilm Carrier

Carrier is an important factor for cell adsorption, immobilization and biofilm formation. The initial adsorption of cells onto the carrier can be caused by the interaction between the cells and the carrier surface, which is associated with the properties of the cells and the carrier (hydrophobicity, specific surface area, porosity, etc.) and environmental cues (pH, temperature, ionic strength, etc.). Various materials such as cotton fibers, wood pulp fibers, bamboo fibers, bricks, bone char, ceramic beads, glass slide, bagasse, linen, silk, synthetic sponge and non-woven fabric have been reported as biofilm carriers for *C. acetobutylicum* (Yen et al., 2011; Dolejš et al., 2014; Zhuang et al., 2017). In other industrial clostridia like *C. tyrobutyricum*, common carriers were also reported. Materials with larger surface area and higher roughness were found better for cells to be adsorbed (Chen et al., 2013). Surface modification of carriers is an important way to improve bacterial adsorption and biofilm formation. For example, polyetherimide and steric acid were used for carrier modification to increase the cations on linen surface and the hydrophobicity of linen surface, respectively. Using the modified linen as a biofilm carrier, the adhesion of *C. acetobutylicum* cells was doubled, and the fermentation yield was also improved (Zhuang et al., 2017).

### Regulatory Genes

Although *C. acetobutylicum* biofilm has been long and extensively applied in solvent production, its genetic mechanisms have been much less studied. Genetic determinants and regulation processes remain largely unknown. Spo0A has been well studied as a master transcription regulator controlling sporulation and solventogenesis. In *B. subtilis*, Spo0A also regulated *epsA-O* genes that were directly responsible for EPS production and biofilm formation (Banse et al., 2011). However, no such genes have been found in *C. acetobutylicum*. In the pathogenic clostridia *C. perfringens*, *spo0A* mutant was not impaired in biofilm formation, whereas in *C. difficile spo0A* mutant showed a defective biofilm formation (Pantaleon et al., 2014). Yang et al. (2020) disrupted the *spo0A* of *C. acetobutylicum* to investigate its influences on biofilm formation for the first time. They found the aggregation and adhesion abilities of the *spo0A* disruptant and its swarming motility were all dramatically reduced compared to those of the wild strain. Wire-like structures that cross-linked cells were also disappeared upon *spo0A* disruption. As a result, the *spo0A* mutant could hardly form biofilm anymore. These phenotypes could be further explained by the proteomic analysis of the *spo0A* mutant. Similarly, disruption of *spo0A* could also decrease biofilm formation in *C. difficile* though the underlying mechanism remained unclear (Dawson et al., 2012). It should be noted that disruption of *spo0A* also severely impaired cell growth and solvent production in *C. acetobutylicum*. So, the abolished biofilm formation upon *spo0A* disruption could potentially be a result of growth defect. More elaborate experiments are therefore needed to dissect the role of Spo0A in biofilm formation in future.

Signaling processes that usually involve kinases-mediated phosphorylation of regulatory proteins, together with intracellular secondary messengers like c-di-GMP or cAMP, are also important for regulation of biofilm formation (O’Toole and Wong, 2016). In *C. acetobutylicum*, CA_C2730 gene encodes a signal transduction histidine kinase (BfcK). There were five orphan histidine kinases and CA_C2730 was the only one not involved in sporulation (Steiner et al., 2011). A recent research demonstrated that CA_C2730 played an important role in the formation of *C. acetobutylicum* biofilm (Liu et al., unpublished). Disruption of CA_C2730 abolished biofilm formation, and flagella were also disappeared in the disruptant with apparently reduced cell motility. Unlike *spo0A*, disruption of CAC2730 did not cause growth defect or impaired fermentation. So, the impact of this gene on biofilm formation would be more plausible. At the same time, phosphorylation proteomics analysis revealed that the presence of CAC2730 probably repressed the phosphorylation of a serine/threonine protein kinase (encoded by CA_C0404) that might negatively regulate biofilm formation. However, these studies are still at the preliminary research stage. More research is needed in the future to understand the molecular basis underlying *C. acetobutylicum* biofilms.

Studies of other clostridia have shown more possible genes involved in biofilm development. In *C. difficile* or *C. perfringens*, a quorum-sensing regulator LuxS, a cell wall protease Cwp84, a germination protein SleC, a signal peptidase SipW, a type IV pilin PilA2 and an EPS matrix protein BsaA have been demonstrated to greatly impact biofilm formation (Ðapa et al., 2013; Obana et al., 2020). Whether homologs of these proteins exist and play similar roles in *C. acetobutylicum* remains to be studied.

## Conclusion and Prospects

*C. acetobutylicum* biofilms have great benefits for industrial applications, but its molecular and genetic basis is still largely unknown. Studies so far have preliminarily characterized the polysaccharide and protein components in the biofilm matrix, studied the cell growth, morphology, metabolism and gene/protein expression pattern in the biofilm, but the studies on biofilm-forming genes and regulatory mechanisms are still insufficient. From our perspective, future research should focus more on the following aspects: (1) understanding the interaction of cells with material surface to better design biofilm-carrying materials; (2) revealing the genetic circuit of biofilm development, and use synthetic biology to re-construct the biosynthetic process for biofilm; (3) exploring the life cycle, physiological changes and clustering effects of the cells in the biofilm under industrial conditions to better develop its potential for industrial applications.

## Author Contributions

HZ, PY, ZW, ML, JZ, DL, YC, and HY contributed to the research, writing, editing, and formatting of the manuscript. All authors contributed to the article and approved the submitted version.

## Conflict of Interest

The authors declare that the research was conducted in the absence of any commercial or financial relationships that could be construed as a potential conflict of interest.
